# Effectiveness of Pfizer Vaccine BNT162b2 Against SARS-CoV-2 in Americans 16 and Older: A Systematic Review

**DOI:** 10.7759/cureus.65111

**Published:** 2024-07-22

**Authors:** Justin Wilburn, Brooke Sappe, Kevin Jorge, Lynn Hickey, Dhatri Nandyala, Tandra Chadha

**Affiliations:** 1 Obesity and Cardiovascular Research, Nemours Children's Health System, Jacksonville, USA; 2 Basic Sciences, Saint James School of Medicine, The Quarter, AIA; 3 Microbiology, Saint James School of Medicine, The Quarter, AIA

**Keywords:** systematic review, united states, vaccine safety, immunogenicity, vaccine, vaccine efficacy, bnt162b2, pfizer, covid-19, sars-cov-2

## Abstract

This systematic review evaluates the efficacy and long-term effectiveness of the Pfizer-BioNTech COVID-19 vaccine (BNT162b2) across diverse clinical and observational settings within the United States in Americans aged 16 and older. We conducted an extensive literature search utilizing various types of studies to assess the vaccine’s performance in preventing symptomatic SARS-CoV-2 infection and severe COVID-19 outcomes. Our initial search in PubMed on March 14, 2022, yielded 6,725 potentially relevant articles, with 26 undergoing full-text assessment and eight meeting the inclusion criteria. To incorporate the most up-to-date findings, a secondary search was conducted on July 6, 2024, using improved and refined Medical Subject Headings (MeSH) terms within the PubMed and Scopus databases. This expanded approach resulted in 78 potentially relevant articles from PubMed and 1,567 from Scopus, with 40 articles undergoing full-text assessment and an additional 14 articles meeting the inclusion criteria. Early clinical trials reported initial vaccine effectiveness (VE) up to 95% with sustained immunity in various populations. Observational studies and systematic reviews further confirmed VE above 90% against symptomatic infections and highlighted nearly complete protection against hospitalizations and deaths. Recent research underscores the critical role of booster doses in maintaining high VE, especially against emerging variants, showing restored effectiveness up to 95% and supporting their strategic importance in ongoing pandemic responses. Despite observed waning immunity and breakthrough infections, the BNT162b2 vaccine continues to exhibit robust protection across different demographic groups and under varying epidemiological conditions. Our findings advocate for continuous booster updates and adaptive vaccination strategies to manage emerging SARS-CoV-2 variants, reinforcing the pivotal role of mRNA vaccine technology in addressing global health emergencies.

## Introduction and background

The emergence of the novel coronavirus SARS-CoV-2 in late 2019 in Wuhan, China, led to the global COVID-19 pandemic. This highly contagious virus has had a profound impact on global health, with infection outcomes ranging from mild respiratory symptoms to severe pneumonia, acute respiratory distress syndrome, and death [1]. By March 11, 2020, the World Health Organization (WHO) officially declared COVID-19 a pandemic, as the virus spread rapidly across continents, resulting in significant morbidity and mortality worldwide [1,2]. 

In response to this unprecedented public health crisis, the development of effective vaccines became a global priority. The Pfizer-BioNTech COVID-19 vaccine (BNT162b2) was among the first to receive emergency use authorization, marking a milestone in vaccine development due to its rapid creation and deployment [3]. Traditionally, vaccine development spans decades, yet the BNT162b2 vaccine was developed and authorized within a year, underscoring the urgency and scientific advancements in the face of the pandemic [3,4]. 

BNT162b2 is an mRNA-based vaccine encapsulated in lipid nanoparticles, designed to elicit an immune response by encoding the SARS-CoV-2 spike protein [3,5]. Clinical trials have demonstrated that two doses of 30 μg each generate robust neutralizing antibody responses and cellular immunity, providing significant protection against COVID-19 [3]. Initial phase three trials reported a vaccine effectiveness (VE) rate of approximately 95% in preventing symptomatic COVID-19, positioning the Pfizer-BioNTech vaccine as a critical tool in controlling the pandemic [5]. 

Despite promising early trial results, observational studies have shown variability in VE due to factors such as emerging variants, population demographics, and timing of vaccine and booster administration [6]. Understanding the vaccine’s performance is essential for effective public health strategies. This study aims to assess the effectiveness of the BNT162b2 vaccine in preventing SARS-CoV-2 infections among Americans aged 16 and older. This article was previously presented as a meeting abstract at the Summer 2022 Saint James School of Medicine Research Day on April 8, 2022.

## Review

Materials and methods 

Search Strategy 

To systematically assess the effectiveness of the BNT162b2 vaccine in Americans aged 16 and older, a search strategy focused on retrieving peer-reviewed articles from PubMed (MEDLINE) and Scopus was used. A combination of Medical Subject Headings (MeSH) terms was utilized across two distinct searches (Table 1). Given the novel emergence of COVID-19 in late 2019, the search was not restricted to a specific start date. The search was intended to encompass all relevant literature from the onset of the pandemic through to the present. Only articles published in English were considered for review.

**Table 1:** Database search queries

Database	Search Query
PubMed (search #1)	(("SARS-CoV-2"[All Fields] OR "COVID-19"[All Fields]) AND "Pfizer vaccine"[All Fields] AND "reinfection"[All Fields]) OR ("COVID-19"[All Fields] AND "vaccine"[All Fields] AND "efficacy"[All Fields])
PubMed (search #2)	("SARS-CoV-2"[All Fields] OR "COVID-19"[All Fields]) AND ("Pfizer vaccine"[All Fields] OR "BNT162b2"[All Fields]) AND ("Pfizer"[All Fields] AND ("vaccine"[All Fields] OR "BNT162b2 vaccine"[All Fields]) AND "reinfection"[All Fields]) AND ("efficacy"[All Fields] OR "effectiveness"[All Fields])
Scopus	(ALL ("SARS-Cov-2" OR "COVID-19") AND ALL ("Pfizer vaccine" OR "BNT162b2 vaccine" AND "reinfection") AND ALL ("Efficacy" OR "effectiveness"))

Inclusion and Exclusion Criteria 

Our systematic review was meticulous in selecting articles that could provide robust insights into the effectiveness of the BNT162b2 vaccine. Articles included in the review reported on individuals aged 16 and older who were diagnosed with or hospitalized due to SARS-CoV-2 infection after receiving both doses of the BNT162b2 vaccine. This inclusion was applied irrespective of the administration schedule of the doses, including cases where the second dose was delayed. Studies that did not align with our research focus or demographic parameters were excluded (Table 2).

**Table 2:** Exclusion criteria applied to searches VE, vaccine effectiveness

Entry	Reason excluded
#1	Non-English
#2	Animal study
#3	Study conducted exclusively outside of the United States
#4	Non-article or review (Letter, Note, Editorial, etc.)
#5	Irrelevant to study aim (adolescents, not vaccine-related, etc.)
#6	Exclusively studying immunocompromised individuals or those with autoimmune diseases
#7	Beyond the scope of VE

SARS-CoV-2 Infection 

The criteria for SARS-CoV-2 infection were participants who had at least two of the following new symptoms: chills, headache, muscle pain, sore throat, any taste or olfactory disorder, fever (temperature ≥ 38°C), cough, shortness of breath, or pneumonia [1]. In addition, participants are required to have one positive test for SARS-CoV-2 through the nucleic acid amplification-based test, such as reverse-transcriptase polymerase chain reaction (RT-PCR) tested on saliva sample, nasal swab, or nasopharyngeal swab [7].

Results 

Study Selection 

The study selection process was structured to ensure comprehensive coverage of relevant literature on the effectiveness of the BNT162b2 vaccine. Initially, a database search in PubMed was conducted on March 14, 2022, yielding 6,725 potentially relevant articles, with 26 undergoing full-text assessment and eight meeting inclusion criteria. This search was designed to capture the breadth of existing research early in the pandemic. To incorporate the most up-to-date findings, a secondary search was conducted on July 6, 2024, using improved and refined MeSH terms within the PubMed and Scopus databases. This expanded approach resulted in 78 potentially relevant articles from PubMed and 1,567 from Scopus, with 40 articles undergoing full-text assessment. An additional 14 articles met the inclusion criteria, greatly broadening the review’s scope and depth. 

**Figure 1:**
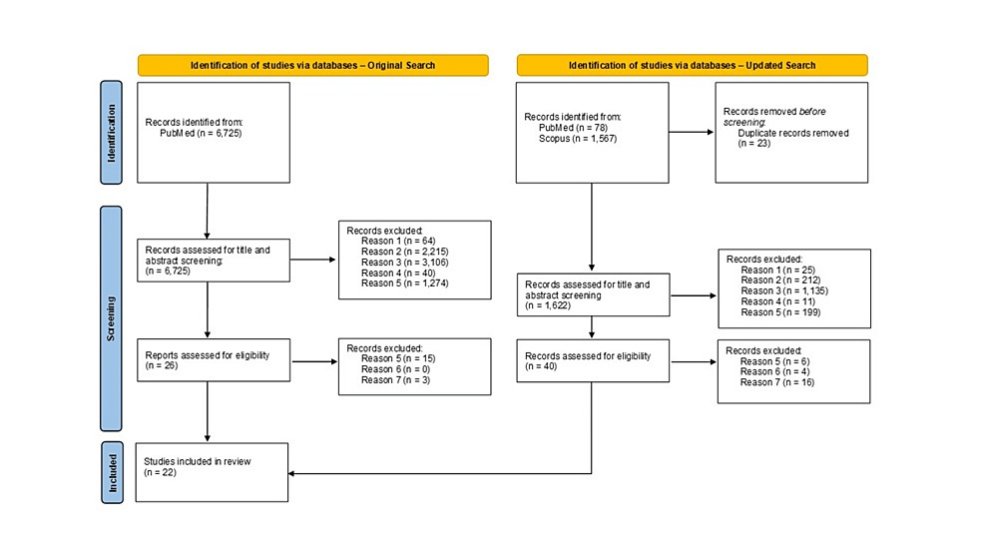
PRISMA flowchart of literature search and article selection process PRISMA, Preferred Reporting Items for Systematic Reviews and Meta-Analyses

The study selection process was illustrated using the Preferred Reporting Items for Systematic Reviews and Meta-Analyses (PRISMA) flowchart, detailing the identification, screening, eligibility, and inclusion of studies (Figure 1). Key findings from the studies selected through this rigorous process are summarized and highlighted (Table 3).

**Table 3:** Characteristics of studies included for review N/A, not applicable; VE, vaccine effectiveness

	Author	Study type	Cohort size	Results
1	Polack et al., 2020 [8]	Double-blind randomized control trial	37,706 participants	52% VE after the first dose and 95% after the second dose
2	Walsh et al., 2020 [9]	Placebo-controlled, observer-blinded, dose-escalation trial	195 participants	Robust immune response with a favorable safety profile across both younger and older adults
3	Kow and Hasan, 2021 [10]	Meta-analysis	37,320 participants	53% VE after the first dose and 95% after the second dose
4	Dooling et al., 2021 [11]	Systematic review	N/A; 30 articles included	91.1% VE against symptomatic COVID-19, 100% against hospitalizations, and 83.3% against COVID-19-related deaths
5	Lutz et al., 2023 [12]	Negative case-control	474 participants	VE of 94.6% against hospitalizations and 86.5% for outpatient visits among American Indian and Alaska Native populations
6	Tenforde et al., 2023 [13]	Case-control	10,078 participants	VE peaked at 92% after 74 days and then decreased to 75% at 270 days post-vaccination
7	Gharpure et al., 2022 [14]	Observational	1,128 participants	55% of breakthrough cases had received BNT162b2, emphasizing the importance of preventive measures in high-transmission settings
8	Garcia-Beltran et al., 2021 [15]	Cross-sectional	99 participants	Stronger neutralization titers in fully vaccinated individuals, highlighting the need for two doses
9	Fraley et al., 2021 [16]	Clinical trial	194 participants	Seropositive individuals showed higher antibody levels after one dose compared to seronegative individuals after two doses
10	Plumb et al., 2024 [17]	Case-control	3,647 participants	VE peaked at 54.8% within the first 59 days following a bivalent dose and then declined to 21.6% after 60 days
11	Plumb et al., 2023 [18]	Case-control	7,277 participants	VE of the BNT162b2 booster peaked at 88.0% within 60 days, which then reduced to 32.1% at 120 days or more during the Omicron variant predominance
12	Shrestha et al., 2023 [19]	Retrospective cohort	51,017 participants	29% VE against Omicron BA.4/5, 20% against BQ lineages, and 4% against XBB lineages
13	Dieckhaus et al., 2023 [20]	Longitudinal cohort	296 participants	Significant decline in neutralizing antibody levels nine months post-vaccination and BNT162b2 neutralization rates of 20% compared to 69% for those vaccinated with mRNA-1273
14	Brunner et al., 2022 [21]	Comparative cohort	647 participants	Higher median antibody titers and greater pseudoneutralization percentages for mRNA-1273 compared to BNT162b2 but it was noted that both vaccines maintained high overall effectiveness
15	Moghadas et al., 2021 [22]	Agent-based modeling	N/A	Delaying the second dose by up to 12 weeks could reduce hospitalizations and deaths if first-dose efficacy remains stable
16	Townsend et al., 2023 [23]	Comparative observational	4,324 participants	Boosting every 6 months to 3 years showed declining probabilities of preventing infection over a 6-year span
17	Hogan et al., 2023 [24]	Transmission modeling	N/A	Demonstrated the effectiveness of biannual booster interventions for high-risk populations, significantly reducing morbidity and mortality
18	Moriera et al., 2022 [25]	Placebo-controlled, randomized, phase III trial	10,125 participants	A third 30 μg dose of the BNT162b2 vaccine achieved a 95.3% VE against the Delta variant
19	Tartof et al., 2023 [26]	Negative case-control	123,419 participants	BNT162b2 BA.4/5 bivalent booster maintained a 56% VE against hospital admissions for XBB-related infections
20	Petrie et al., 2023 [27]	Prospective longitudinal cohort	883 participants	The initial BNT162b2 booster had a 74% VE in the first 90 days against Omicron sublineages, which waned to 36% after 6 months, though a second booster increased protection by 24% in older adults
21	Song et al., 2023 [28]	Systematic review and meta-analysis	N/A; 42 articles included	Reported that a first booster enhanced protection significantly, showing 53.1% VE against infection and 82.5% against severe outcomes
22	Andrejko et al., 2023 [29]	Matched, negative case-control	2,238 participants	Found that a booster dose increased VE from 53.2% to 95% against symptomatic infections

Discussion 

*Summary of Key Findings* 

**Figure 2:**
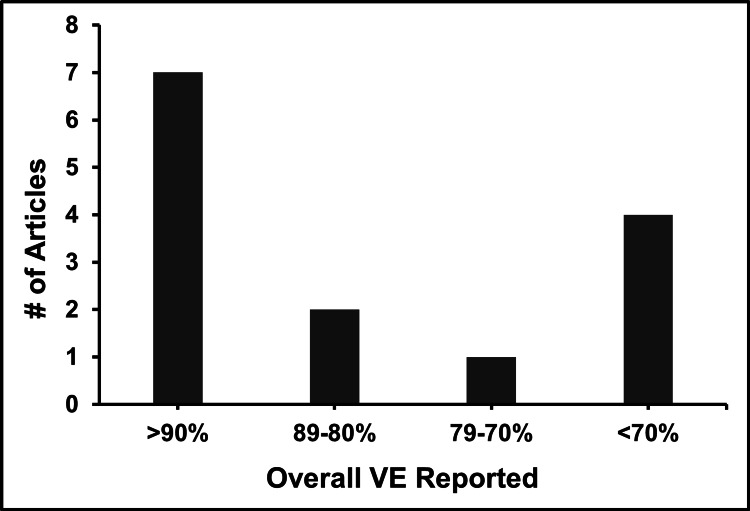
Distribution of VE reported in reviewed articles VE, vaccine effectiveness

The comprehensive analysis of the BNT162b2 vaccine across various studies highlights its robust effectiveness in clinical and observational settings. Initial clinical trials demonstrated that BNT162b2 elicited a strong immune response and a high safety profile, with VE reaching up to 95% against symptomatic SARS-CoV-2 infections. Subsequent observational studies and systematic reviews have confirmed sustained high effectiveness in broader populations, demonstrating over 90% VE in preventing symptomatic infections and nearly complete protection against COVID-19-related hospitalizations and deaths. More recent findings emphasize the importance of booster doses, which significantly restore and sustain VE against emerging variants and breakthrough infections. Collectively, the findings in this review validate the continued efficacy of the BNT162b2 vaccine, reinforcing the critical role of booster doses in long-term defense against the virus across diverse demographic and clinical contexts. The distribution of VE as reported across different studies is visually summarized in Figure 2, which categorizes the studies based on their reported VE percentages.

VE in Clinical and Observational Settings

In early evaluations of the BNT162b2 vaccine, the double-blind, randomized controlled study by Polack et al. demonstrated a VE of 95% for preventing symptomatic SARS-CoV-2 infection among participants aged 16 and older [8]. This study, which involved a one-to-one random assignment to receive either the vaccine or a placebo with doses administered 21 days apart, showed significant protection starting 12 days after the first dose and substantial protection seven days following the second dose [8]. Concurrently, the placebo-controlled, observer-blinded dose-escalation study by Walsh et al. assessed the vaccine’s safety and immunogenicity across two age groups (18-55 and 65-85 years) with varied dosage levels (10, 20, 30, and 100 μg) administered at a 21-day interval [9]. The findings from this study highlighted the vaccine’s capacity to induce strong SARS-CoV-2 neutralizing geometric mean titers, which were comparable to or higher than those observed in convalescent serum samples, underscoring the vaccine’s potent immune response. Notably, the vaccine showed a favorable balance of reactogenicity and immunogenicity, particularly in older adults, where it prompted lower incidence and severity of systemic reactions compared to the earlier BNT162b1 candidate [9]. Findings from Polack et al. and Walsh et al. were instrumental in advancing BNT162b2 into later-phase trials, highlighting its strong immune response and favorable safety profile across diverse age groups [8,9].

In conjunction with the controlled trials, early observational analyses and systematic reviews further elucidated the effectiveness of the BNT162b2 vaccine across broader population settings, though these studies also noted some variability due to emerging variants and diverse vaccination protocols. The meta-analysis conducted by Kow and Hasan, which pooled data from 19 observational studies, utilized hazard ratios (HR) and incidence rate ratios (IRR) to measure VE [10]. Their analysis revealed a 53% VE after the first dose and a 95% VE after the second dose, as evidenced by RT-PCR tests conducted at least seven days post-vaccination [10]. Similarly, the systematic review by Dooling et al. provided a comprehensive evaluation of the vaccine’s performance, synthesizing data from three phase II/III clinical trials and 22 observational studies [11]. This review confirmed the VE of BNT162b2 in preventing symptomatic, laboratory-confirmed COVID-19 at 91.1% from clinical evidence and 92.4% from observational studies. It also highlighted a 100% effectiveness in preventing COVID-19-associated hospitalizations and 83.3% in preventing COVID-19-related deaths from clinical data, with observational evidence supporting a VE of 94.3% for hospitalizations and 96.1% for deaths. Furthermore, observational studies showed 89.3% effectiveness in preventing asymptomatic SARS-CoV-2 infections, emphasizing the vaccine’s extensive protective capabilities across diverse demographic settings [11].

Recent studies have demonstrated the sustained effectiveness of BNT162b2 against hospitalizations, even amid the emergence of variants like Omicron. Lutz et al. found high VE among American Indian and Alaska Native (AI/AN) populations, with 233 (78.2%) receiving BNT162b2 [12]. The study did not differentiate VE between the Pfizer and Moderna vaccines, but collectively, they exhibited a VE of 94.6% in preventing COVID-19-associated hospitalizations and 86.5% for outpatient visits among AI/AN individuals, highlighting their robust protective impact [12]. Tenforde et al. assessed VE among all immunocompetent adults, observing that overall BNT162b2 VE peaked at 92% after 74 days and decreased to 75% at 270 days post-vaccination [13]. In contrast, VE for mRNA-1273 peaked at 94% after 83 days and decreased to 86% by day 270. A noticeable waning effect was evident among those vaccinated with BNT162b2, with VE decreasing from 88% to 79% for adults aged 18-64 and from 87% to 78% in those aged 65 and older. While the waning effect was greater for older adults, the level of protection remained comparably robust across all age groups, highlighting the vaccine’s sustained efficacy [13].

Emerging variants of concern (VOCs) have also prompted the need for further insight into breakthrough cases. Despite a high vaccination rate among their cohort, Gharpure et al. identified 1,128 cluster-associated COVID-19 cases, 918 (81%) being breakthrough infections in fully vaccinated individuals who recently participated in large public gatherings [14]. Notably, 504 (55%) breakthrough cases occurred in individuals who received the BNT162b2 vaccine, 293 (32%) with the mRNA-1273 vaccine, and 121 (13%) with the Janssen Ad26.COV2.S (Johnson & Johnson) vaccine. These findings further support the significant waning immunity and breakthrough infection susceptibility among those vaccinated with BNT162b2 against highly transmissible VOCs [14].

*Neutralization and Immune Response* 

Garcia-Beltran et al. investigated the neutralization efficacy of sera from individuals vaccinated with either BNT162b2 or mRNA-1273 against pseudoviruses representing 10 globally circulating strains of SARS-CoV-2 [15]. The study found that variants harboring receptor-binding domain mutations, such as P.1 and B.1.351, exhibited significantly reduced neutralization even in fully vaccinated individuals. The cohort, with a median age of 33 years, included sera from 99 vaccinated individuals compared to 1,220 pre-pandemic samples. The pseudovirus neutralization assay demonstrated high sensitivity (100%) and specificity (99%) for discriminating vaccinated individuals >7 days post-second dose. Notably, individuals receiving both doses of the BNT162b2 vaccine (>7 days apart) exhibited stronger neutralization titers. These findings highlight the critical need for two full doses of the vaccine to achieve adequate serum titers and suggest the potential requirement for updated vaccine formulations to address emerging variants [15].

In the clinical trial by Fraley et al., the humoral immune responses during SARS-CoV-2 mRNA vaccine administration were characterized in both seropositive and seronegative individuals [16]. The study involved healthcare workers who received the BNT162b2 vaccine, with antibody levels measured before and after each dose. The findings revealed that seropositive individuals exhibited significantly higher antibody levels after a single vaccine dose compared to seronegative individuals who required two doses to reach similar antibody titers. Specifically, IgG, IgA, and IgM responses were assessed using multiplex bead-based assays targeting various SARS-CoV-2 antigens, and neutralizing antibodies were evaluated using a surrogate virus neutralization test. The results demonstrated that vaccination elicited robust IgG responses in both groups, but only seronegative individuals showed significant increases in IgA and IgM levels post-vaccination. Additionally, the study mapped antibody epitope specificity, identifying key immunodominant peptides on the SARS-CoV-2 spike protein targeted by antibodies after immunization. These findings underscore the strong immune response elicited by the BNT162b2 vaccine, particularly in individuals with prior SARS-CoV-2 infection, and highlight the potential for using prior infection history to guide vaccination strategies [16].

Effectiveness Among Healthcare Workers

Vaccination among healthcare workers is paramount, serving as a critical line of defense in preventing the transmission of infectious diseases within medical settings. Ensuring high VE is essential, as it not only protects healthcare professionals but also enables them to safely care for vulnerable patients without the risk of spreading COVID-19. In recent studies, Plumb et al. (2023) demonstrated the effectiveness of the BNT162b2 vaccine among healthcare workers [17]. Within the first 59 days following a bivalent dose of BNT162b2, peak effectiveness reached 54.8%, further waning to 21.6% at 60 days or more post-vaccination. Despite this decline, the overall effectiveness of the cohort was recorded at 34.0%, slightly lower than the mRNA-1273 vaccine’s 34.6% [17]. 

Further research from Plumb et al. (2023) revealed a significant boost in VE post-booster administration during the Delta variant predominance, with the Pfizer booster achieving an effectiveness of 88.0% within 60 days [18]. This effectiveness was reduced to 73.4% in less than 60 days post-booster and further dropped to 32.1% at 120 days or more during the Omicron variant predominance. Despite these variances, the overall adjusted VE of the Pfizer booster was marked at 71.2%, showcasing the critical role booster doses play in maintaining a robust immunogenic response, irrespective of the original or booster vaccine brand [18]. 

In a study involving 51,017 healthcare personnel employed with the Cleveland Clinic, Shrestha et al. evaluated the effectiveness of the bivalent COVID-19 vaccines [19]. Although VE was not individually assessed for the BNT162b2 and Moderna mRNA-1273 vaccines, 87% of the vaccinated subgroup, which constituted only 26% of the total cohort, received BNT162b2. The VE was 29% against Omicron BA.4/5 lineages, dropped to 20% for BQ lineages, and fell to 4% against XBB lineages, indicating variable protection that diminished with emerging variants [19]. 

In a longitudinal observational study at UConn Health, Dieckhaus et al. found that healthcare workers vaccinated with the BNT162b2 vaccine showed a significant decline in neutralizing antibody levels nine months post-vaccination compared to those vaccinated with the mRNA-1273 vaccine, with neutralization rates of 20% versus 69%, respectively [20]. Additionally, median antibody titers for BNT162b2 declined sharply from 3.29 μg/mL at two months to 0.65 μg/mL at nine months, a more pronounced reduction than observed in Moderna recipients (3.47 μg/mL at two months to 2.07 μg/mL at nine months) [20]. Similar trends were observed by Brunner et al. (2022), who reported higher median antibody titers and greater pseudoneutralization percentages in recipients of the mRNA-1273 vaccine [21]. Despite the more robust and durable immune response elicited by mRNA-1273 compared to BNT162b2, both vaccines maintained high overall effectiveness: 89% for Moderna and 87% for Pfizer. Moreover, the administration of booster doses significantly amplified immunogenic responses, regardless of whether the booster was homologous or heterologous to the initial vaccine series [21]. This strategy underscores the critical role of timely boosters in extending VE, which is vital for safeguarding both healthcare workers and their patients against COVID-19 exposure. 

Proposed Vaccination Strategies and Booster Doses

Additional vaccination strategies have been proposed, such as those by Moghadas et al., who investigated the impact of delaying the second dose of vaccines, including the BNT162b2 vaccine [22]. Using an agent-based model of COVID-19 transmission, the study compared the epidemiological impact of the standard two-dose schedule with a delayed second dose (DSD) strategy. The results indicated that delaying the second dose of the BNT162b2 vaccine by up to 12 weeks could reduce hospitalizations and deaths, provided the efficacy of the first dose does not wane significantly over time. Specifically, a nine-week delay in administering the second dose could avert an additional 0.60 hospitalizations and 0.32 deaths per 10,000 population compared to the standard three-week schedule [22]. 

While initial vaccination strategies, such as those explored by Moghadas et al., suggest potential benefits from delaying the second dose of vaccines like BNT162b2 to reduce hospitalizations and deaths, this approach hinges critically on the sustained efficacy of the initial dose [22]. As we consider the longevity of vaccine-induced immunity, further exploration into booster doses becomes essential. Townsend et al. provide crucial insights into this aspect by projecting the longevity of antibody levels and the corresponding effectiveness against infection over extended periods with different booster frequencies [23]. Their study shows that boosting every six months to three years yields declining probabilities of fending off infection over a six-year span, with rates of >89%, 69%, 49%, 36%, and 23% for BNT162b2 [23]. 

Expanding on these considerations, the modeling study by Hogan et al. emphasizes the nuanced approach required in booster dose strategies to effectively mitigate the long-term impact of SARS-CoV-2 transmission [24]. Their analysis suggests that targeted booster interventions, especially for high-risk populations such as those aged 75 and older in high-income countries, can significantly reduce morbidity and mortality, averting up to 1,770 hospitalizations and 531 deaths per million population with biannual boosting. Regularly scheduled boosters, particularly with vaccines adapted to current variants, were shown to nearly double the prevention of hospitalizations and deaths compared to ancestral vaccines. Furthermore, the study underscores the cost-effectiveness of these strategies, noting that annually updated vaccines could enhance health outcomes, with the cost per hospitalization averted up to $7,048 and the cost per death averted up to $30,065. This aligns with the need for a dynamic vaccination strategy that can respond to the ongoing challenges posed by new VOCs and waning immunity over time [24]. 

Building on the strategic importance of booster doses in maintaining long-term protection against SARS-CoV-2, a range of empirical studies, including clinical trials and observational analyses, further substantiates their efficacy. For example, early research into booster administration provides compelling data on their impact. A placebo-controlled, randomized trial by Moreira et al. demonstrated that administering a third 30 μg dose of the BNT162b2 vaccine achieved 95.3% efficacy against the Delta variant within their cohort, with the third dose given a median of 10.8 months after the second dose [25]. Complementing these findings, a study by Wiemken et al. provides robust evidence on the broader implications of booster vaccinations among U.S. adults [26]. The study revealed that individuals who received booster doses were 25% less likely to test positive for COVID-19, exhibited a 14% reduction in the risk of developing long COVID, and experienced significantly milder symptoms, highlighting the booster’s comprehensive benefits in enhancing protection and reducing the severity and long-term impacts of the virus [25]. 

Further emphasizing the importance of booster doses, recent studies document their sustained efficacy across various SARS-CoV-2 variants and critical health outcomes. Tartof et al. highlighted that the BNT162b2 BA.4/5 bivalent booster maintained a 56% VE against hospital admissions for XBB-related infections, demonstrating its robustness against severe COVID-19 outcomes [26]. Similarly, Petrie et al. found that while the initial BNT162b2 booster showed a 74% VE in the first 90 days against Omicron sublineages BA.1 through BA.5, its effectiveness waned to 36% after six months, though a second booster increased protection by 24% in older adults [27]. Adding to this, Song et al. reported that a first booster enhanced protection significantly, showing 53.1% effectiveness against infection and 82.5% against severe outcomes, with this protection enduring over time: 77.6% and 85.9% for first and second boosters, respectively [28]. Andrejko et al. reinforced these findings by demonstrating that a booster dose not only increased VE from 53.2% to 95% against symptomatic infections but also confirmed through bias-corrected methodologies that maintaining such high effectiveness is crucial for long-term defense against the virus, strongly advocating for ongoing booster administration [29]. 

Despite the rapid timeline for BNT126b2 production and manufacturing, the high effectiveness is promising with regard to future vaccine development technology [30]. The success of the BNT162b2 vaccine and subsequent booster doses have demonstrated the potential of mRNA vaccine technology, paving the way for rapid responses to future public health crises. The lessons learned from the development and deployment of this vaccine could accelerate the creation of vaccines for other infectious diseases [30,31].

Study limitations 

This systematic review has several limitations. The included studies exhibit significant heterogeneity in design, population characteristics, and outcome measures, complicating direct comparisons and synthesis of results. The geographical focus on the United States limits the generalizability of findings to other regions with different demographic and healthcare profiles. Additionally, the rapid evolution of SARS-CoV-2 and the emergence of new variants may not be fully represented in the current literature, potentially affecting the applicability of our findings to ongoing pandemic dynamics. Despite these limitations, the review is strengthened by a methodological approach that provides a synthesis of data across various populations and settings. This allows for valuable insights into the long-term effectiveness of the BNT162b2 vaccine, particularly highlighting the crucial role of booster doses in sustaining immunity against emerging variants. The inclusion of studies examining the impact of booster doses has demonstrated their ability to restore and maintain high levels of VE, even as the virus evolves. This aspect is particularly vital for informing adaptive public health policies and vaccination strategies, ensuring they remain effective in the face of changing viral dynamics and ongoing challenges posed by new VOCs.

Future directions 

Ongoing research is crucial to assess the BNT162b2 vaccine’s effectiveness against emerging SARS-CoV-2 VOCs [31]. Future studies should focus on the long-term immunity afforded by booster doses and evaluate the vaccine’s efficacy in immunocompromised and autoimmune populations to optimize vaccination strategies. Additionally, adapting vaccine formulations to address variant-related changes and integrating new data into public health strategies will be essential for maintaining the efficacy of vaccination efforts globally, especially in reducing severe disease outcomes.

## Conclusions

The Pfizer-BioNTech COVID-19 vaccine, BNT162b2, has demonstrated high effectiveness in preventing SARS-CoV-2 infection among Americans aged 16 and older, achieving VE rates up to 95% against symptomatic COVID-19 across diverse settings. Our systematic review has underscored the vaccine’s robust performance in reducing severe outcomes like hospitalizations and deaths, even though VE varies due to factors such as emerging variants, demographic differences, and timing of vaccinations. The importance of booster doses has been particularly highlighted, showing their crucial role in maintaining high VE amid waning immunity and the emergence of new variants. Moreover, the success of BNT162b2 illustrates the potential of mRNA technology for rapid vaccine development, providing a robust platform for responding to future public health crises effectively. 

Challenges in generalizing the findings globally due to the U.S.-centric nature of the studies call for extended global research and adaptive vaccination strategies. Future studies should also explore the long-term immunity afforded by booster doses and assess the vaccine’s efficacy in diverse and immunocompromised populations.
